# Molecular Characteristics of Bean Common Mosaic Virus Occurring in Inner Mongolia, China

**DOI:** 10.3390/genes15010133

**Published:** 2024-01-21

**Authors:** Jingru Li, Zhengnan Li, Zhanmin Wu, Yu Sun, Suqing Niu, Mengze Guo, Lei Zhang

**Affiliations:** 1 College of Horticulture and Plant Protection, Inner Mongolia Agricultural University, Hohhot 010018, Chinalizhengnan@imau.edu.cn (Z.L.);; 2 Ordos Center of Agriculture and Animal Husbandry Ecology and Resource Protection, Ordos 017000, China; 3 Inner Mongolia Academy of Agricultural and Animal Husbandry Sciences, Hohhot 010010, China

**Keywords:** potyvirus, genome, phylogeny, recombination analysis

## Abstract

Bean common mosaic virus (BCMV) was detected on common bean (*Phaseolus vulgaris*) plants showing wrinkled and/or narrow leaves, curling, shrinking and chlorosis of leaves, dwarfing of plants, and mottled pods in Inner Mongolia and named BCMV-22Huhe. Its genome has a size of 10,062 bp and was deposited in GenBank under the accession number OR778613. It is closely related to BCMV-Az (GenBank accession no. KP903372, in China) in the lineage of AzBMV. A recombination event was detected for BCMV-22Huhe among the 99 BCMV isolates published in the NCBI GenBank database, showing that BCMV-CJ25 (MK069986, found in Mexico) was a potential major parent, and the minor parent is unknown. This work is the first description of the occurrence of BCMV in Inner Mongolia, China.

## 1. Introduction

Bean common mosaic virus (BCMV) is a member of the genus *Potyvirus* [1], it has a genome of a single-stranded RNA molecule of about 10,000 nucleotides. BCMV is widely distributed worldwide, and yield loss due to BCMV can be as high as 100% [2,3,4]. It is a potential threat to the bean industry in China [5]. BCMV can be transmitted through various ways, including mechanical inoculation, seeds, pollen grains, and aphids [6,7].

Common bean (*P. vulgaris*) is herbaceous annual plant grown worldwide for its edible dry seeds or green, unripe pods, and it is known by many different names, including French beans, string beans, and snap beans. The world production of string beans was about 1.3 million tons from about 0.14 million ha in 2021 (https://www.fao.org/home/en, accessed on 21 December 2023). Inner Mongolia is one of the main production areas for common beans in China. With an increasing cultivation area and continuous cropping for years, disease and pest problems have become increasingly prominent, especially the occurrence of viral diseases [5,8]. In recent years, mosaic, wrinkling and other viral diseases of beans have been observed on land and in greenhouses in Inner Mongolia, affecting the yield and quality of beans and limiting the sustainable development of the bean industry. Here, we described the molecular characteristics of BCMV occurring on common bean in Inner Mongolia, China.

## 2. Materials and Methods

### 2.1. Virus Source

**Figure 1:**
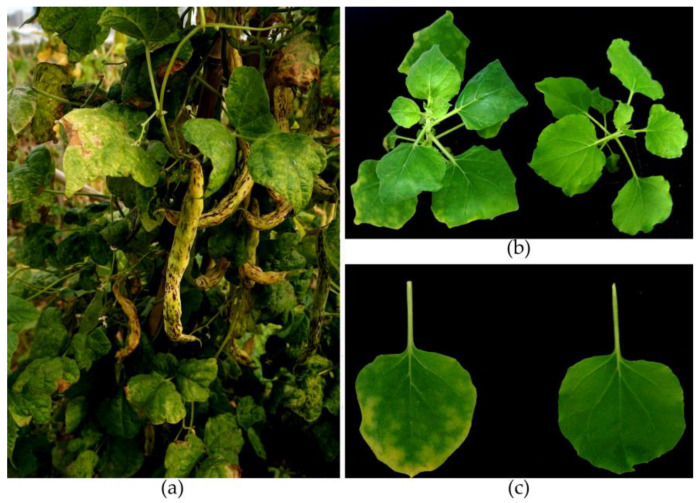
BCMV-infected common bean and *Nicotiana benthamiana* plants. (**a**) A common bean plant with wrinkled leaves, chlorosis of leaves, and mottled pods. (**b**,**c**) Symptoms caused by BCMV-22Huhe on *N. benthamiana* plants eight days post inoculation.

In 2022, common bean (*P. vulgaris*) plants showing wrinkled leaves, chlorosis of leaves, and mottled or deformed pods (Figure 1a) were observed in Hohhot, Inner Mongolia, China, suggesting viral infection. A visual incidence rate of about 50% occurred for the disease. Samples of fresh leaves of the diseased common bean plants were collected for pathogen diagnosis.

### 2.2. Amplification of the Genomic Fragments of BCMV

The total RNA of each sample was extracted using a Spectrum^TM^ plant total RNA kit (Sigma-Aldric, St. Louis, MO, USA), and its concentration was determined using a micro-spectrophotometer, a Nano-300 (Allsheng, Hangzhou, China). The 5′ and 3′ termini of the BCMV isolate were amplified using a SMARTer RACE kit (Takara, Dalian, China), following the manufacturer’s instructions. The middle part of the BCMV isolate was cloned in 3 fragments (Table 1). Briefly, using the RNA samples as templates, cDNAs were synthesized using a PrimeScript II 1st strand cDNA synthesis kit (Promega Biotechnology, Beijing, China) following the manufacturer’s instructions. The cDNAs served as templates in PCR assays for amplifying the genomic cDNA fragments of BCMV. The PCR reaction mixture (20 µL) was prepared as follows: cDNA, 2.0 µL; M5 superlight mix (Mei5 Biotechnology, Beijing, China), 10 µL; nuclease-free H_2_O, 6.0 µL; forward and reverse primers, 1.0 µL each. The primers (Table 1) were designed based on the conserved parts of the complete genome sequences of 96 isolates of BCMV deposited in the NCBI GenBank database. The PCR program was set as follows: pre-denaturation at 95 °C for 3 min, 35 cycles of 30 s at 94 °C, 30 s at Tm (Table 1), and 1 kb/min at 72 °C, and a final extension of 10 min at 72 °C. 

### 2.3. Cloning and Sequencing of the BCMV Fragments

The PCR products were separated on 1% agarose gel, and the fragments of expected sizes were purified using a Tiangen Midi purification kit (Tiangen, Beijing). The purified fragments were inserted into a pTOPO-TA cloning vector (Aidlab Biotechnologies, Beijing). The ligation mixture included 5 µL of the purified fragments, 1 µL of pTOPO-TA Vector, 1 µL of 10× Enhancer, and 3 µL of nuclease-free H_2_O, and the mixture was incubated 2 h at 37 °C. The ligation product was used for the transformation of *Escherichia coli* JM109 competent cells. Positive colonies were screened using a colony PCR, and the positive ones were sent to Sangon Biotech (Beijing) for Sanger sequencing.

### 2.4. BCMV Genome Assembly and Sequence Analysis

The obtained query sequences were submitted to the BLASTn online tool to retrieve homologous sequences from the NCBI GenBank database, and the sequences, verified BCMV genome fragments, were assembled using Vector NTI 11.5 (Invitrogen, Carlsbad, CA, USA), generating the full-length genome of the BCMV isolated in Hohhot, named BCMV-22Huhe. 

Based on the complete nucleotide sequences of BCMV-22Huhe and the 17 representative strains of BCMV, including strains Az (KP903372), soybean (KC832501), Taiwan (AY575773), R (NC_003397), NL1 (AY112735), NL4 (DQ666332), blotch (U05771), PStV (U34972), JX014 (KJ807813), OR_C (KF919298), P (KF919300), DXH025 (KJ807812), HZZB007 (KJ807814), US1 (KT175569), US-10 (KF919299), Ir_GoB (MF498886), and CJ25 (MK069986) [6,9], a phylogenetic tree was reconstructed using MEGA11 [10] via the maximum likelihood method (general time-reversible model + γ distributed with invariant sites) with bootstrap replicates of 1000, whereas the complete genome sequence of Zucchini yellow mosaic virus (ZYMV) (NC_003224) was employed as an outgroup reference. 

A recombination analysis was conducted for BCMV-22Huhe and the 99 BCMV isolates (including the 15 representative strains) and published in the NCBI GenBank database using the Recombination Detection Program 4 (RDP4) software, in which seven different methods were used for the recombination analysis, namely RDP, GENECO NV, BOOTSCAN, MAXCHI, CHIMAERA, SISCAN, and 3SEQ. With *p* ≤ 0.05 as a standard [11], if more than three methods were detected at the same time, it was judged to be a meaningful recombination event.

### 2.5. BCMV Host Range 

To identity potential host plants for BCMV, healthy plants of seven different species were sap-inoculated using the juice of the BCMV 22Huhe-infected common beans. The tested plants included *N. benthamiana*, *N. occidentalis*, *N. tabacum*, *N. glutinosa*, *Chenopodium quinoa*, *C. amaranticolor*, and *Datura stramonium*. The inoculated plants were maintained in a glasshouse under 16/8 h of light and darkness at 20–25 °C and 60% humidity. The inoculated plants were observed daily for possible symptom development. Eight days post inoculation, an RT-PCR was performed to detect BCMV in the inoculated plants.

## 3. Results

### 3.1. Genome Structure and Sequence Identity Analysis of BCMV Isolates

The complete genome of BCMV-22Huhe consists of 10,062 nucleotides. Through alignment mainly with the representative strain of BCMV-R in Zhejiang, China (GenBank accession no. NC_003397), it was annotated and encodes a large open reading frame (ORF), expressing a polymeric protein of about 358 kDa which can be spliced into 10 functional proteins, i.e., P1, HC-Pro, P3, 6K1, CI, 6K2, NIa-VPg, NIa-Pro, NIb, CP, and a small frameshift ORF from the P3 gene, producing PIPO. The genome of BCMV-22Huhe had nucleotide sequence identities of 81.7–93.8% to the genomes of the 99 BCMV isolates published in NCBI GenBank database, while their amino acid sequence identities ranged from 87.0% to 96.3%. Moreover, BCMV-22Huhe has the highest nucleotide sequence identity with BCMV-C54 and -313615 (GenBank accession no. OP828732 in China and MH024840 in the USA), and the lowest nucleotide sequence identity with BCMV-RU1P (KF919300 in the USA); the same is true for its amino acid sequence identity. 

### 3.2. Phylogeny and Recombination Analyses of BCMV Isolates

**Figure 2:**
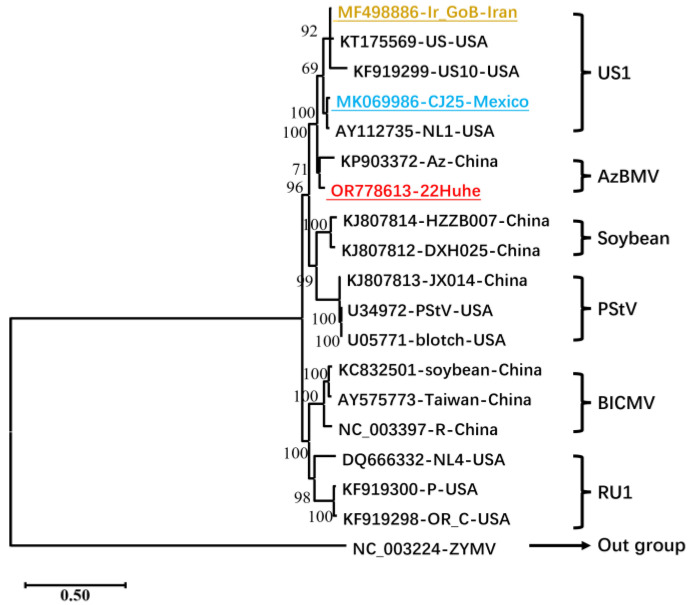
The phylogenetic tree based on the whole-genome sequences of BCMV. The tree was constructed using the maximum likelihood method (general time-reversible model + GI). GenBank accession numbers, strains, and countries are labeled for each node; BCMIV-22Huhe in this work is in red, its potential major parent of CJ25 is in blue, and a possibly potential minor parent of Ir_GoB is in brown. Closing braces show the lineage of related clades.

To determine the classification of BCMV-22Huhe, a phylogenetic tree including 17 representative strains of BCMV was constructed based on the complete genome sequences of the BCMV isolates (Figure 2). On the tree, BCMV 22Huhe was closely related to BCMV-Az (KP903372), suggesting the AzBMV lineage of BCMV-22Huhe. 

**Figure 3:**
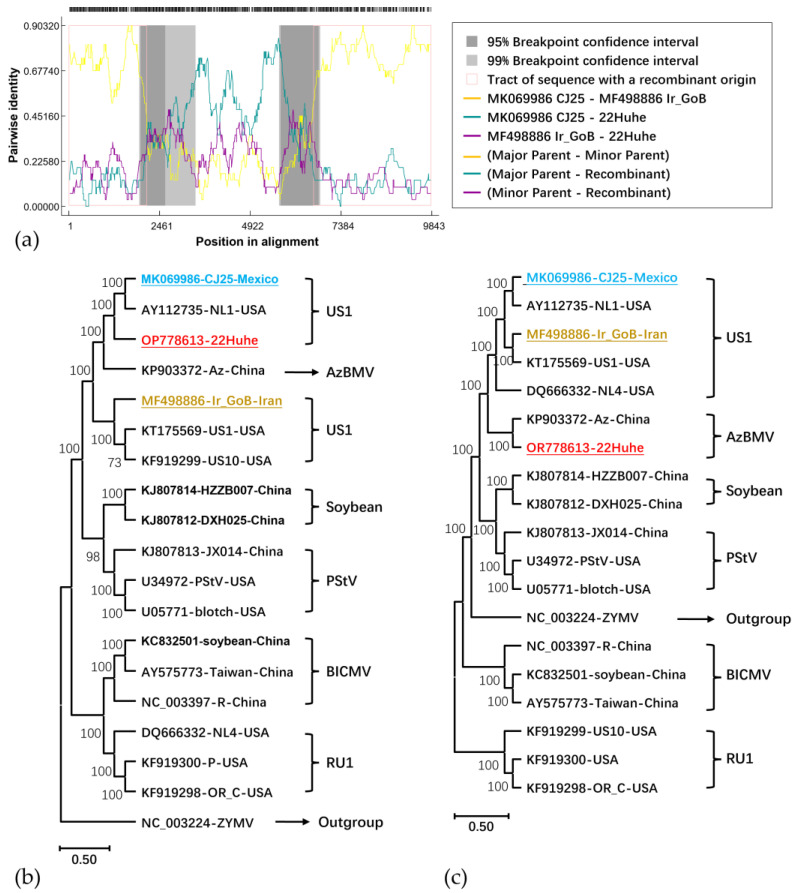
Recombination analysis for BCMV-22Huhe. (**a**) The result of an RDP analysis of a recombination event in 22Huhe using RDP4; (**b**) the maximum likelihood phylogenetic tree based on recombinant sequences; (**c**) the maximum likelihood phylogenetic tree based on the assembled sequence of non-recombinant sequences. The trees were constructed using a bootstrapping method with 1000 replications. BCMIV-22Huhe in this work is in red, its potential major parent of CJ25 is in blue, and a possibly potential minor parent of Ir_GoB is in brown.

Among the 99 BCMV isolates published in the NCBI GenBank database, a recombination event was detected for BCMV-22Huhe which was supported by all seven algorithms (Table 2). The recombination site was found from 2121 nt (99% confidence interval 1903 nt–3443 nt) to 6626 nt (99% confidence interval 5717–6824 nt); the major parent was BCMV-CJ25 (MK069986 found in Mexico), while the minor parent was unknown. BCMV-IrGoB (MF498886 found in Iran) was suggested as a possibly potential minor parent (Figure 3a). Using the recombinant fragment of the representative strains, a phylogenetic tree (Figure 3b) was rebuilt on which BCMV-22HuHe was closely related to the potential major parent, BCMV-CJ25 (MK069986), in the US1 lineage. This supported the result of the recombination analysis. While removing the recombinant sequences from the complete genome and assembling the non-recombinant sequences for constructing another phylogenetic tree, on the tree, BCMV-22HuHe was found to be closely related to BCMV-Az (KP903372) in the AzBMV lineage (Figure 3c), in agreement with the result of the phylogenetic analysis using the completed genome sequences above.

### 3.3. BCMV Host Range and Symptoms

In a total, of 42 plants of seven different species were sap-inoculated using the infected common bean plants, that is, *N. benthamiana*, *N. occidentalis*, *N. tabacum*, *N. glutinosa*, *C. quinoa*, *C. amaranticolor*, and *D. stramonium* were inoculated. Eight days after inoculation, only the *N. benthamiana* plants developed symptoms of mosaic and wrinkled leaves (Figure 1b,c). All plants were detected for BCMV-22Huhe infection using an RT-PCR with the primers BCMV-F/-R; the conditions were set as described above. A fragment of about 490 bp was amplified from the symptomatic *N. benthamiana*, and the sequences were corrected. No amplicons were seen from the other tested plants.

## 4. Discussion

BCMV exists as a complex of strains exhibiting significant biological and genetic diversity. Several host-related lineages of BCMV were defined, with US1/NL1 and RU1 originating in common bean [12,13], PStV in peanut [5,14], BlCMV in blackeye cowpea [5,15], one in soybean [5], and one in azuki bean [16,17]. The occurrence of BCMV in China, however, was known until very recently. From 2014 to 2023, different BCMV isolates were reported infecting diverse plant species in China, for example, BCMV-HB (KC478389) infected mungbean in Jiangsu Province in 2014 [18], BCMV isolate closely related to the BCMV-NL1 (KM023744) infected common bean in Liaoning in 2018 [19], BCMV-DHJ1 infected *Polygonatum kingianum* in Yunnan Province in 2019 [20], BCMV SY-Peanut (MN786956) infected peanut in Liaoning Province in 2020 [19], BCMB-CT (KM076650) infected bamboo in Jiangsu Province in 2022 [21], BCMB-Az (KP903372) infected rice bean in Beijing in 2023 [22], and BCMV-NKY021 (KJ807819) infected yam bean in Zhejiang Province in 2023 [23]. BCMV-22Huhe showed nucleotide sequences identities of 90.1–99.0% to the five BCMV isolates above. In addition, many complete genome sequences of BCMV isolates in China were published in the NCBI GenBank database. A phylogenetic analysis based on the complete genome sequences of these isolates revealed that most of them were placed in the lineage of soybean, and there were fewer isolates in the lineage of AzBMV; the ones we knew were BCMV-Az (KP903372 from Beijing, China) and BCMV-NTZ1 (MZ670770 from Zhejiang, China). Here, BCMV-22Huhe might be another isolate in China that can be placed in the lineage of AzBMV.

Recombination was a key source of emerging genetic variation [24]. It was proposed that recombination was a relatively common process in potyviruses, and recombination was usually parented by isolates from the same geographical region as the recombinant [25,26]. In addition, it was claimed that to a large extent, the diversity in BCMV populations was driven by both host species and geographic location [27,28]. Using RDP4 software, a major parent of BCMV-CJ25 (MK069986) which infected *P. vulgaris* in Mexico was detected for BCMV-22Huhe; the minor parent, however, was unknown. Given the international trade of plant seeds or plant materials, it was not surprising to find the spread of viruses among different geographic locations, even among countries, seeing CJ25 as a potential major parent of 22Huhe in China. It was remarkable that no potential minor parent was detected for BCMV-22Huhe among the 99 published isolates. We hypothesized that specific genetic variations may exist in Inner Mongolia. Considering that both BCMV-22Huhe and -CJ25 occurred on *P. vulgaris*, in comparison with geographical factors, host-driven factors may have a limit role in the formation of the non-recombinant part of BCMV-22Huhe, while geographically specific variants somewhere may have donated their genetic sequences as a minor parent. This is clearly an area that needs further attention. This work was the first description of the occurrence and characteristics of BCMV isolated in Inner Mongolia, China.

## Figures and Tables

**Table 1:** Primers used for the detection and amplification of the full-length genome of the BCMV.

Primer	Sequences (5′-3′)	Tm/°C	Amplicon Size/bp	Primer Location/bp
BCMV-F1	CTAACTGTGGTCGGTTCACCCA	60	3870	253–4060
BCMV-R1	CCCTACTTGGTGGTGGTTGGA	60		253–4060
BCMV-F2	ATGAACTTTCCACAGCTTCTGTAGTGAG	60	3942	5875–9817
BCMV-R2	TTATGGAGAGCATCACTGTAGGGTGC	62		5875–9817
BCMV-F3	TATAAAGTTTCTCGTCTTCCTTCCCAT	58	2812	3807–6619
BCMV-R3	CAACTGCGAAGTCACACCTCAGAAG	61		3807–6619
5′ RACE	GCTTTTGATTTCTATGAGGTGA	57	9252	610–9862
3′ RACE	CTAGGTTTGAAGAGGAGGTGCG	59		610–9862
BCMV-F	ACAACACACTCATGGTTGTAAT	60	490	1256–1746
BCMV-R	CTGATTCTGCTATGTACGGAGC	66		1256–1746

**Table 2:** Recombination analysis for BCMV-22Huhe using RDP4.

Methods	*p*-Value
RDP	9.216 × 10^−10^
GENECONV	8.188 × 10^−11^
BlootScan	6.100 × 10^−20^
MaxChi	1.347 × 10^−18^
Chimaera	9.245 × 10^−7^
SiScan	1.803 × 10^−16^
3Seq	7.209 × 10^−8^

## Data Availability

The sequence data were submitted to GenBank databases under the accession number OR778613. The address is as follows: GenBank http://www.ncbi.nlm.nih.gov (accessed on 18 January 2024).
